# Impact of *ZBTB7A* hypomethylation and expression patterns on treatment response to hydroxyurea

**DOI:** 10.1186/s40246-018-0177-z

**Published:** 2018-10-01

**Authors:** Vasiliki Chondrou, Eleana F. Stavrou, Georgios Markopoulos, Alexandra Kouraklis-Symeonidis, Vasilios Fotopoulos, Argiris Symeonidis, Efthymia Vlachaki, Panagiota Chalkia, George P. Patrinos, Adamantia Papachatzopoulou, Argyro Sgourou

**Affiliations:** 1 School of Science and Technology, Biology Laboratory, Hellenic Open University, Patras, Greece; 2 Faculty of Medicine, Biology Laboratory, University of Ioannina, Ioannina, Greece; 3 Thalassemia and Hemoglobinopathies Unit, Hematology Division, Department of Internal Medicine, General University Hospital of Patras, Patras, Greece; 4 School of Science and Technology, Digital Systems and Media Computing Laboratory, Hellenic Open University, Patras, Greece; 5 Medical School, Hematology Division, Department of Internal Medicine, University of Patras, Patras, Greece; 6 Thalassemia Unit, “Hippokrateio” General Hospital of Thessaloniki, Thessaloniki, Greece; 7 Thalassemia and Sickle Cell Unit, AHEPA University General Hospital of Thessaloniki, Thessaloniki, Greece; 8 School of Health Sciences, Department of Pharmacy, Laboratory of Pharmacogenomics and Individualized Therapy, University of Patras, Patras, Greece; 9 Medical Faculty, Laboratory of General Biology, University of Patras, Patras, Greece

**Keywords:** SCA homozygotes, SCA/β-thal heterozygotes, Hydroxyurea treatment, HbF induction, *HBB* cluster “modifying genes”, Epigenetic regulation

## Abstract

**Background:**

We aimed to clarify the emerging epigenetic landscape in a group of genes classified as “modifier genes” of the β-type globin genes (*HBB* cluster), known to operate *in trans* to accomplish the two natural developmental switches in globin expression, from embryonic to fetal during the first trimester of conception and from fetal to adult around the time of birth. The epigenetic alterations were determined in adult sickle cell anemia (SCA) homozygotes and SCA/β-thalassemia compound heterozygotes of Greek origin, who are under hydroxyurea (HU) treatment. Patients were distinguished in HU responders and HU non-responders (those not benefited from the HU) and both, and in vivo and in vitro approaches were implemented.

**Results:**

We examined the CpG islands’ DNA methylation profile of *BCL11A*, *KLF1*, *MYB*, *MAP3K5*, *SIN3A*, *ZBTB7A*, and *GATA2*, along with γ-globin and LRF/*ZBTB7A* expression levels. In vitro treatment of hematopoietic stem cells (HSCs) with HU induced a significant DNA hypomethylation pattern in *ZBTB7A* (p*, 0.04) and *GATA2* (p*, 0.03) CpGs exclusively in the HU non-responders. Also, this group of patients exhibited significantly elevated baseline methylation patterns in *ZBTB7A*, before the HU treatment, compared to HU responders (p*, 0.019) and to control group of healthy individuals (p*, 0.021), which resembles a potential epigenetic barrier for the γ-globin expression. γ-Globin expression in vitro matched with detected HbF levels during patients’ monitoring tests (in vivo) under HU treatment, implying a good reproducibility of the in vitro HU epigenetic effect. LRF/*ZBTB7A* expression was elevated only in the HU non-responders under the influence of HU.

**Conclusions:**

This is one of the very first pharmacoepigenomic studies indicating that the hypomethylation of *ZBTB7A* during HU treatment enhances the LRF expression, which by its turn suppresses the HbF resumption in the HU non-responders. Its role as an epigenetic regulator of hemoglobin switching is also supported by the wide distribution of *ZBTB7A*-binding sites within the 5′ CpG sequences of all studied human *HBB* cluster “modifier genes.” Also, the baseline methylation level of selective CpGs in *ZBTB7A* and *GATA2* could be an indicator of the negative HU response among the β-type hemoglobinopathy patients.

**Electronic supplementary material:**

The online version of this article (10.1186/s40246-018-0177-z) contains supplementary material, which is available to authorized users.

## Introduction

Thalassemias and sickle cell anemia (SCA) are still of the most common genetic disorders among human population even after many decades of worldwide prenatal control attempts. Furthermore, the combination of β^s^ allele, as a consequence of the SCA genotype with β-thalassemic alleles (SCA/β-thal), appears with an evenly severe clinical phenotype. Clinical practice has shown that, despite identical disease-causing mutations, the pathological phenotype of disease varies enormously between individual patients. This is partly related to genetic variants of the genetic loci coding for the hemoglobin subunits alpha and beta (*HBA* and *HBB*) including their remote regulatory regions (LCRs—locus control regions) [1, 2] and the co-interaction with other parameters such as epigenetic regulatory mechanisms [3].

These genetic disorders have an early onset, within a few months after birth, which is accompanied by high financial burdens to healthcare systems due to the expensive therapeutic protocols applied to patients. Apart from the regular blood transfusions and iron-chelating agents, an alternative widely used therapeutic approach is the induction of fetal hemoglobin (HbF) expression that ameliorates their pathological phenotype [4, 5]. Hydroxyurea (HU) or hydroxycarbamide is the major therapeutic agent identified as a potent HbF inducer and has been used for the management of patients with SCA and SCA/β-thal, since its FDA approval in1998 [6, 7]. The main mechanism of HU action is the inhibition of the ribonucleotide reductase (RR), which pauses the DNA synthesis. The in vivo effects of HU in mammalian systems are transient, as a consequence from the rapid absorption, metabolism, and excretion of the drug. Once-daily dosing of HU presumably causes an intermittent cytotoxic effect resulting in stress erythropoiesis and increased HbF levels [8]. Patients administered with HU show elevated HbF levels within the first 3 to 6 months of treatment, with further improvements up to 12 months, where patients reach a plateau with the maximum HbF expression levels and the corresponding maximum mean erythrocyte corpuscular volume (MCV) [9]. However, HbF levels do not present a uniform increase in all patients, whereas long-term use of HU sometimes leads to the loss of its initial beneficial action [10, 11]. The underlying precise molecular mechanism of the HU action and the variance of treatment results remain still elusive, though a concealed epigenetic effect of its action is highly suspected.

Epigenetic modifications, such as DNA methylation and histone methylation/acetylation, constitute an additional layer of gene expression regulation and affect phenotypic heterogeneity of human diseases and drug responses [12, 13]. DNA methylation mainly occurs in the context of CG dinucleotides (CpGs) and has traditionally been associated with gene repression [14].

This study concerns the methylation alterations, induced by HU treatment on known β-type globin genes (*HBB* cluster) “modifier genes”:erythroid-specific transcription factors (*KLF1*, *GATA1*, and *GATA2*), regulators of hematopoiesis and erythropoiesis (*MYB*, *SOX6*, *SIN3A*), members of the MAPK signaling pathway (MAP3K5), and HbF repressors (*BCL11A*, *ZBTB7A*) [15–28], in terms of contributing to knowledge on the molecular epigenetic regulation of embryonic HbF (α_2_γ_2_) reactivation during the human progenitor cells’ exposure to HU. Though *SOX6* and GATA*1* bared no CpG islands within neighboring sequences, indicating that they are alternatively regulated and were excluded from the study, CpG islands’ quantitative analysis of hyper- or hypomethylation status within the *HBB* cluster “modifier genes” has been elaborated both in patients comprised within a long-term treatment and newly introduced to HU. The epigenetic landscape of these genes has also been studied during cultivation and erythroid differentiation of human-induced hematopoietic pluripotent stem cells derived from healthy donors’ and patients’ peripheral blood, cord blood, and bone marrow, under the pressure of HU to simulate in vitro the pharmacological induction of HbF and the corresponding cell molecular responses.

The aim of our study was to depict and highlight changes in the DNA methylation pattern at genomic loci unlinked to the *HBB* cluster on chromosome 11, which may influence the HbF induction in adults, to distinguish the different epigenetic regulatory nuclear mechanisms acting among responders and non-responders to treatment with HU and to contribute to the development of a clinical algorithm for improved prediction of the patients’ response to HU treatment.

## Material and methods

### Bioinformatics analysis and CpG islands’ selection

**Fig. 1:**
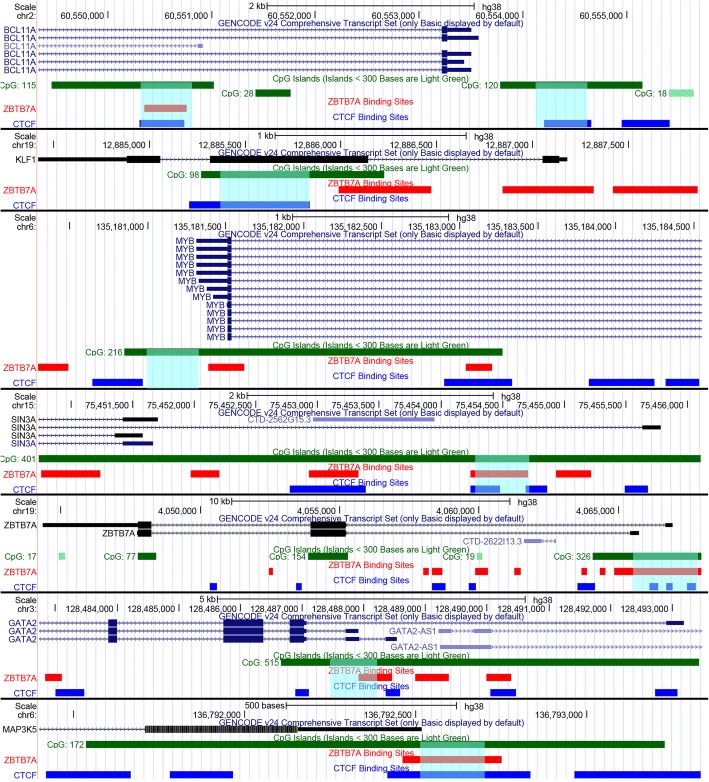
Epigenetic features of promoter regions of genes analyzed in this study. The promoter regions and sequence features of *BCL11A*, *KLF1*, *MYB*, *SIN3A*, *ZBTB7A*, *GATA2*, and *MAP3K5* respectively are presented in parallel tracks, from top to bottom: scale, chromosomal location, gene transcript from Gencode v.24 (exons are represented as boxes and introns as lines with arrows indicating the 5′ towards the 3′ region), CpG islands (boxes in green color), LRF/*ZBTB7A*-binding sites (boxes in red color), and CTCF-binding sites (boxes in blue color). Chromosomal locations of interest in each promoter region are highlighted with light blue transparent boxes

Candidate disease identified “modifier loci,” encoding β-type globin gene regulators were: *BCL11A*, *KLF1*, *MYB*, *MAP3K5*, *SOX6*, *SIN3A*, *ZBTB7A*, *GATA1*, and *GATA2* [15–28]. CpG islands of all genes were retrieved by the UCSC genome browser (genome.ucsc.edu) [29]. Selected CpG islands for the study are located at areas flanking genes’ promoter or within downstream 5′ UTR regions and gene bodies. Respective data for gene locations and CpG islands analyzed are presented in Table 1. All include sites for CTCF and/or ZBTB7A transcription factors’ commitment (Fig. 1). Genes and/or CpG sites that did not preserve these characteristics were excluded from the study.

**Table 1:** Chromosomal locations of all CpG islands analyzed

Gene	Location of CpG island	CTCF-binding site	ZBTB7A-binding site
*BCL11A*	CpG 120	**+**	**–**
	Chr2: 60554555-60554409		
	Chr2: 60554304-60554174		
*BCL11A*	CpG 115	**+**	**+**
	Chr 2: 60550535-60550416		
*KLF1*	CpG 98	**+**	**–**
	Chr 19: 12885764-12885569		
	Chr 19: 12885322-12885477		
*MYB*	CpG 216	**–**	**–**
	Chr 6: 135181253-135181153		
	Chr 6: 13518140-135181107		
*SIN3A*	CpG 401	**+**	**+**
	Chr 15: 75454578-75454439		
*ZBTB7A*	CpG 326	**–**	**+**
	Chr 19: 4067422-4067286		
	Chr 19: 4066671-4066789		
*GATA2*	CpG 515	**–**	**+**
	Chr 3: 128488082-128487980		
*MAP3K5*	CpG 172	**+**	**+**
	Chr 6: 136792589-136792545

Transcription factor binding sites for CTCF and ZBTB7A were extracted from ENCODE project Chip-Seq data [30] and visualized in the UCSC genome browser.

### Sample collection

Patients were selected based on the existence of homozygosity to HbS (SCA) or compound heterozygosity to SCA and β-thalassemia (SCA/β-thal). All patients were adults, either treated for long-term with HU or for immediate treatment initiation, due to life-threatening disease-related symptoms. For the newly introduced patients to HU therapy, the mean dosage of HU administered was 20 mg/kg and the mean follow-up time was 5–6 months. Upon arrival to the plateau phase, where maximum HbF was produced, patients continued to be monitored annually. HbF levels at baseline and 5–6 months after initiation of HU treatment were estimated in all patients participating in this study with the high-performance liquid chromatography (HPLC) method, HbA_2_/HbA_1c_ dual program (VARIANT™ II Beta-Thalassemia, Bio-Rad) (Additional file 1: Figure S1). Patients’ hematological data are presented in Additional file 1: Table S1.

Blood samples of SCA, SCA/β-thal, and healthy donors have been included in the study. Experimental procedures have been applied both in vivo and in vitro.

Samples analyzed in vivo were group I—30 peripheral blood samples from SCA and SCA/β-thal patients, sub-categorized to responders and non-responders to HU (Additional file 1: Table S1), and group II—20 peripheral blood samples from healthy donors to act as reference group.

Samples analyzed in vitro were as follows: Group III—burst-forming unit-erythroid (BFU-E) cell populations from hematopoietic progenitor human cells acquired from the peripheral blood of selected SCA/β-thal patients who were undergoing HU therapy for the first time. Total samples cultivated derived from five responders (III R1–R5) and for non-responders (III NR1–NR4). Their characterization as responsive or not to the HU treatment was done later, when they had reached the plateau phase (5–6 months). III NR3 and NR4 were sisters and brothers. Group IV—BFU-E colonies acquired from peripheral blood of the control group (four samples IV CG1–CG4), from the bone marrow (four samples, IV BM1–BM4), and from the umbilical cord blood (four samples, IV UCB1–UCB4), all coming from healthy donors (Table 2).

**Table 2:** In vitro samples (III and IV)

Sample ID	In vitro γ-globin (+HU/−HU)	Baseline HbF (−HU)	Plateau HbF (+HU)	In vivo HbF (+HU)/(−HU)
IV CG1	1.75	x	x	x
IV CG2	1.38	x	x	x
IV CG3	1.26	x	x	x
IV CG4	1.69	x	x	x
III NR1	1.35	3.4	4.6	1.35
III NR2	1.54	2.5	2.9	1.16
III NR3	2.12	0.6	1	1.67
III NR4	1.43	2.1	2.9	1.38
III R1	1.09	6.1	22.6	3.7
III R2	3.66	6.7	16.4	2.5
III R3	2.31	10.7	31	2.89
III R4	2.56	9.8	24.5	2.5
III R5	1.87	2.7	15.1	5.6
IV UCB1	2.02	x	x	x
IV UCB2	2.57	x	x	x
IV UCB3	1.96	x	x	x
IV UCB4	2.34	x	x	x
IV BM1	5.58	x	x	x
IV BM2	4.44	x	x	x
IV BM3	3.25	x	x	x
IV BM4	5.02	x	x	x

Responders and non-responders to HU were clinically distinguished in relevance to their hematological parameters before and after administration of HU, their transfusion needs, and the capability to ameliorate anemia symptoms. For the present study, which focused on the epigenetic regulatory differences among the *HBB* cluster “modifier genes” and their influence in HbF induction, the strict clinical evaluation of patients has not been applied. Responders were considered the patients with an overall improved disease course and a ≥ 2.5-fold HbF increase.

Hematopoietic stem cells from categories III and IV have been cultivated simultaneously in the presence and absence of HU (50–100 μΜ).

### Hematopoietic pluripotent stem cell cultures

**Fig. 2:**
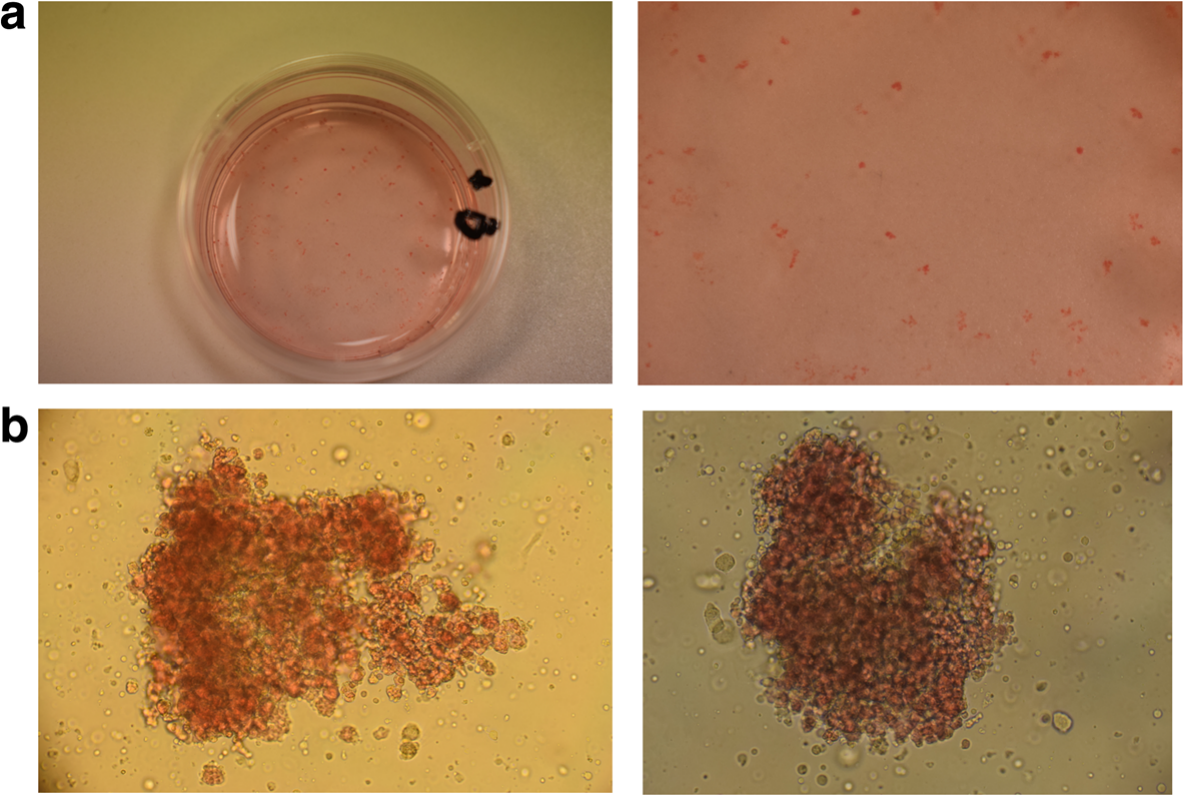
BFU-E colonies derived from CFU assay. **a** Plate with BFU-E colonies, at day 16 (end of culture period), naturally colored red due to hemoglobin synthesis. **b** Two representative BFU-E colonies, observed under the inverted microscope (× 100)

The hematopoietic pluripotent stem cells derived from human blood samples support an in vitro clonogenic culture system, known as colony-forming unit assay (CFU), simulating the in vivo erythropoiesis in the bone marrow. The cell colonies of the different morphology finally obtained by this type of cultures are the CFU-E (colony-forming unit-erythroid) and the BFU-E (burst-forming unit-erythroid), which are apparent after 14–16 days of continuous culture (Fig. 2).

Peripheral blood (PBMCs) and cord blood mononuclear cells, enriched in CD34^+^, were isolated with Histopaque-1077 (Sigma-Aldrich) following the vendor’s protocol. Single cell common myeloid progenitors (isolated from bone marrow aspirates) were also induced for erythrocyte expansion and differentiation in the same culture conditions. In all cases, 10^5^ cells were cultivated in the semi-solid culture media (MethoCultt GF+ H4435, StemCell Technologies), containing the appropriate cytokine cocktail plus erythropoietin.

At day 13, culture medium was supplemented with the pharmacological factor HU (Sigma), freshly prepared, at concentrations between 50 and 100 mM for monitoring the effect of the pharmaceutical agent. In parallel, CFUs were carried under the same conditions apart from the addition of HU. At day 16, when cells reached the BFU-E stage of differentiation, cultures were terminated. Viability of the cells was checked by trypan blue dye exclusion test. Single BFU-E colonies were randomly picked under an inverted microscope (Leica DMI), whereas the rest of the colonies (derived from the same plating) were collectively pooled and used for DNA/RNA extraction. Genetic material from BFU-Es was processed to bisulphite treatment followed by pyrosequencing methylation assays and RNA to qPCR experiments.

### Detection of relative gene expression by RNA quantification (qRT-PCR)

In quantitative PCRs, the amount of target cDNA (either γ-globin or LRF/*ZBTB7A*) was compared and normalized to the amount of *GAPDH* reference gene expression. cDNAs were amplified using QuantiFast SYBR Green PCR (Qiagen) and the ECO (Illumina) instrument. The target and internal reference gene were run in separate duplicate or triplicate reactions using primers reported at Table 3. γ-Globin, towards *GAPDH* reaction conditions, were as follows: 10 s at 95 °C, 30 s at 58 °C, 40 cycles (Additional file 1: Figure S1), and LRF/*ZBTB7A* to *GAPDH* were done according to Shen et al. [31] (Fig. 5).

**Table 3:** Pyrosequencing and qPCR primer sets

Gene	CpG islands	Sequence	Primers
*BCL11A*	CpG 120	5′ GTTGTTTTGGAGTGGGAG 3′	Forward
		5′ CAAATTAAAAACTAAACCTCCAAATTAC 3′	Reverse 5′-biotin labeled
		5′ TTTTTTTTTTGAGATTTTTAGGAGT 3′	Forward/Pyrosequencing
		5′ATTACTCCCCAACACCCTCCT 3′	Reverse 5′-biotin labeled
		5′ TTTTATTTTTTTTAGTTAGTTG 3′	Pyrosequencing
	CpG 115	5′ GTTAGTTTGGGAGGGGGTGA 3′	Forward
		5′ AAACCAAATACAAACTTACCATATCC 3′	Reverse 5′-biotin labeled
		5′ GTTATGTGGGTTGAATG 3′	Pyrosequencing
*KLF1*	CpG 98	5′ GGTTAGGGGTTGGTGGTTGG 3′	Forward
		5′ AACCCCCCCCCCTCACCTATA 3′	Reverse 5′-biotin labeled
		5′ TGGGTTTAGTTTTGGTTTTA 3′	Pyrosequencing
		5′ GAGGATTTAGGTGTGA 3′	Pyrosequencing
*MYB*	CpG 216	5′ GATAGTGAGTGGGAGTTGGAGGA 3′	Forward
		5′ CCATCAAACAAAAAACTTTAAACACT 3′	Reverse 5′-biotin labeled
		5′ GGAGGAGAGAGAGTAGAATGGGAG 3′	Pyrosequencing
		5′ GGTTTGTTTAGGAAAAGG 3′	Pyrosequencing
*SIN3A*	CpG 401	5′ AGGGGGTGGTTTGAAAGG 3′	Forward
		5′ ATATCATCCAATCCACATCCAAAA 3′	Reverse 5′-biotin labeled
		5′ GGGAAAAGGAAATGTATTAG 3′	Pyrosequencing
*ZBTB7A*	CpG 326	5′ GTAGATTTTTTTGTGTTAAGGA 3′	Forward
		5′ AACAAACCCCCAACCTCTAC 3′	Reverse 5′-biotin labeled
		5′ GGGATTTTTATAGTTTTATTTTTAA 3′	Pyrosequencing
		5′ GGGTTTTGGTTGTATTGTATAGTTAT 3′	Forward
		5′ CTCATACACTTAACCCCCAAT 3′	Reverse 5′-biotin labeled
		5′ GAGGGAGAGATTAGGGTA 3′	Pyrosequencing
*GATA2*	CpG 515	5′ ATTGTTAGGGAGGTTTAGAGTAT 3′	Forward
		5′ ACTCTCAAACCCCAAACTT 3′	Reverse 5′-biotin labeled
		5′ GAGAGAGTAGGGAGGGGGT 3′	Pyrosequencing
*MAP3K5*	CpG 172	5′ GTGGTGGAGAGGGAGAGAGTTTGTAAG 3′	Forward
		5′ CTAACCAACCACAACTCCAAACTACTCC 3′	Reverse 5′-biotin labeled
		5′ GTTTTTTTTGGTTTTTTTTAGA 3′	Pyrosequencing
qPCR primer sets			
*HBG*	–	5′-GAC AAG CTG CAT GTG GAT CCT-3′	Forward
		5′-CCG AAA TGG ATT GCC AAA AC-3′	Reverse
*ZBTB7A*	–	5′-CAT CTG CGA GAA GGT CAT CC-3′	Forward
		5′-TGT CCT GCC TGG TGA AGC-3′	Reverse
*GAPDH*	–	5′-CCA TGT TCG TCA TGG GTG TGA-3′	Forward
		5′CAT GGA CTG TGG TCA TGA GT-3′	Reverse

Gene expression in BFU-E colonies was calculated as relative expression of treated compared to untreated with HU colonies, using the formula 2^(−ΔCt)^ and normalized to the reference *GAPDH* gene.

### Methylation analysis

The methylation status of all CpG DNA sequences was determined with the Pyrosequencing CpG assay methodology in duplicate or triplicate analysis indicating a good technical reproducibility and validation of the method. To achieve the complete conversion of the unmethylated cytosines (existing as CpG dinucleotides) to uraciles, bisulphite method (Qiagen) was utilized. Pyrosequencing reactions were performed with the PyroMark Q24 MDx technology (Qiagen). Methylated and unmethylated (bisulphite converted) human DNAs plus unmethylated (physiological) human DNA were used as internal controls in every round of pyrosequencing reactions. Different sequencing primers, specific for the methylation analysis of each DNA CpG sequence, were designed as well as all PCR primers sets with the PyroMark Assay Design Software, version 2.0 (Qiagen) (Table 3). Variation in methylation status of various CpG sites was detected and presented in a sequence context (Additional file 1: Figure S2, S3). Quality controls for the bisulphite conversion efficiency were introduced in every Pyrosequencing run set.

### Sodium bisulphite treatment modification for the single-colony CpG assays

Before DNA methylation analysis, normal cytosine residues, existing as CpG dinucleotides, were completely converted to uracil residues while 5-methylocytosine residues remained unchanged through sodium bisulphite conversion reaction. In this study, a modified protocol for bisulphite treatment of single colonies of human erythroid progenitor cells was developed bypassing the DNA extraction step and based on the standard protocol of EpiTect Bisulphite kit (Qiagen, Hilden, Germany) for sodium bisulphite conversion of unmethylated cytosines in DNA from low-concentration solutions. In particular, for the preparation of bisulphite reactions, a single colony of each sample (of maximum 10^6^ cells per colony) was stored at − 80 °C for at least 2 h to obtain cell membrane damage. After thawing, 40 μl of DNase-free water was added, mixed by vigorous pipetting, and the protocol for the low concentration solutions of DNA was followed. The eluted (treated) DNA per single colony was stored at − 20 °C until use.

### Statistical analysis and correlation of methylation and expression results

Statistical analysis was performed for all data collected regarding percentage of methylation per CpG site and mRNA levels. Results were correlated either to the corresponding gene expression percentages or to the methylation/hypomethylation impact of the CpG islands of all studied genes before and after the HU treatment.

The results of the methylation analysis were presented as mean ratio of the methylation levels at CpG sites within gene loci of interest. Statistical analysis and boxplots were performed using SPSS software version 20. Depending on the normality tests of datasets, independent sample *t* test or Mann-Whitney test was used to compare untreated (−HU) single BFU-E colonies and single BFU-E colonies treated with HU (+HU) between each group. Also, the one-way ANOVA or the Kruskal-Wallis test for not normally distributed data was used for the comparison of methylation status between both untreated single BFU-E colonies and in vivo samples in all subgroups.

In all cases, *p* values less than 0.05 were considered statistically significant.

## Results

### Detection and recording of γ-globin expression (in vitro) in the presence of hydroxyurea

Total RNA collected from CFU cultures was subjected to a quantitative PCR (qPCR) assay to detect γ-globin mRNA expression profile. Variation in γ-globin levels were observed in biological replicate experiments with CFU cultures from the same donor, which prompted us to refine the HU concentrations used in culture conditions (50–100 μM). Finally, culture media was supplemented with the maximum cell-tolerated HU dose (100 μM) and subsequently the effects on γ-globin gene expression were normalized, to wit γ-globin level variations between sister CFU cultures from the same donor were of no statistical significance.

BFU-E cell colonies grown at 100 μΜ HU showed, with no exception, an increase in γ-globin calculated as relative expression. Non-responders (III NR1–4) showed a maximum of 2.1-fold increase while responders (III R1–5) a 3.66-fold, all compared to their related untreated cells (−HU) (Additional file 1: Figure S1). BFU-Es from healthy donors (IV CG1–4, UCB1–4, and BM1–4) showed a 1.3–5.6-fold increase in γ-globin expression.

### Correlation of γ-globin induction (in vitro) with HbF levels (in vivo) upon HU treatment

The elevated γ-globin gene expression obtained from the ex vivo culture system (CFU) was compared to the corresponding in vivo HbF levels determined among patients (responders and non-responders) at relevant time points, namely, at baseline, before HU treatment, and at the plateau phase (after 5–6 months of continuous HU treatment). HbF levels were detected during regular preset patients’ hematological tests at the Hematology Division of the Patras’ University Hospital with the HPLC method. Responders (III R1–5) showed augmented HbF percentage, within a range of 2.5–5.6-fold, while non-responders (III NR1–4) exhibited an increased HbF percentage, always lower than 2.5-fold difference (1.1–1.7) from baseline. Increased γ-globin gene expression (ex vivo) under the presence of HU was well correlated with HbF expression levels in patients at the plateau phase (in vivo) confirming experimental culture conditions and concentrations of HU added (Table 2).

### In vivo methylation levels within CpG islands of *HBB* cluster “modifier genes” among non-responders and responders receiving HU treatment and control group

Methylation profiles of the selected CpGs were tested in genomic material derived from peripheral mononuclear blood cells (PBMCs) of non-responders (I NR1–11) and responders (I R1–19) under long and continuous HU therapy, and of the control group (II CG1–20) (Additional file 1: Table S1). CpGs tested were CpG120 and CpG115 of *BCL11A*, CpG98 of *KLF1*, CpG216 of *MYB*, CpG172 of *MAP3K5*, CpG401 of *SIN3A*, CpG326 of *ZBTB7A*, and CpG515 of *GATA2* (Table 1).

DNA from PBMCs showed an extensive heterogeneity of the methylation levels of CpG islands, and non-significant results were recorded apart from the *SIN3A* CpG 401. *SIN3A* CpG 401 was deferentially methylated between non-responders (I NR1–11) and responders (I R1–19) (p*, 0.024), with the non-responders exhibiting higher methylation rates across the entire CpG DNA sequence (data not shown). Since it is not a tissue-specific factor [32], the statistically significant differentiation of *SIN3A* methylation with a consequent differential expression of its protein product within PBMCs was not an unexpected result.

*KLF1* CpG98, *ZBTB7A* CpG326, and *GATA2* CpG515 did not alter significantly their methylation levels compared to all subgroups, before and after HU treatment. *MYB* CpG216, *MAP3K5* CpG172, and *BCL11A* CpG120 had an established intrinsic methylation level of less than 10% among all groups; therefore, we concluded that we could not deliver reliable results of the HU effect on their methylation status.

### Differentiation of methylation profiles of *HBB* cluster “modifier genes” caused by HU treatment in single BFU-E colonies derived from non-responders, responders, and control groups

To further explore the potential epigenetic influence of HU and to particularize it on cells of the erythroid lineage, we isolated well-defined single BFU-E colonies (screened under an inverted microscope) [33] and determined hyper-/hypomethylation status of all CpGs in every single colony based on a slightly diverged bisulphite conversion protocol (described in the “Material and methods” section) before and after the HU treatment of the cells. Although an inter-individual character between BFU-E-matured cells within a single colony could not be excluded, uniform distribution of methylation profiles was observed in experimental replicates of individual samples and among two or three (2–3) different BFU-E colonies tested from the same donor.

Regarding the healthy control group, pluripotent hematopoietic stem cells from the peripheral (IV CG1-CG4) and umbilical cord blood (IV UCB1-UCB4) as well as from bone marrow aspirations (IV BM1-BM4) were included, to explore possible fundamental epigenetic similarities with hematopoietic pluripotent stem cells derived from the patient’s groups (III NR1-NR4 and III R1-R5). Responders (III R1-R5) and non-responders (III NR1-NR4) were also included in the in vitro assays, but had not entered the HU treatment regimen yet (in vitro samples, Table 2). Assortment of Rs and NRs was done long after the HU treatment initiation, based on their clinical characteristics and HbF induction at plateau phase. Hematopoietic pluripotent stem cells from all different tissues were expanded as described in the “Material and methods” section both without (−HU) and with (+HU) HU addition (100 μM), and at the BFU-E stage, two to three single colonies from every sample were isolated separately and subjected to methylation assay.

The methylation status of all *HBB* cluster “modifier genes” showed mean values below 50–60% along the entire CpG sequences and exhibited hypomethylation in the presence of HU among all different groups of BFU-Es tested, which is consistent with the results from whole genome methylation assays during cell transition from early hematopoietic CD34^+^ to erythroid lineage committed cells [34].

*MYB* CpG216, *MAP3K5* CpG172, and *BCL11A* CpG120 had the exact negligible methylation level of less than 10% before (−HU) and after the addition of the drug (+HU), as mentioned in the in vivo methylation reactions; therefore, statistical analysis of these areas was not performed either (Additional file 1: Figure S3).

**Fig. 3:**
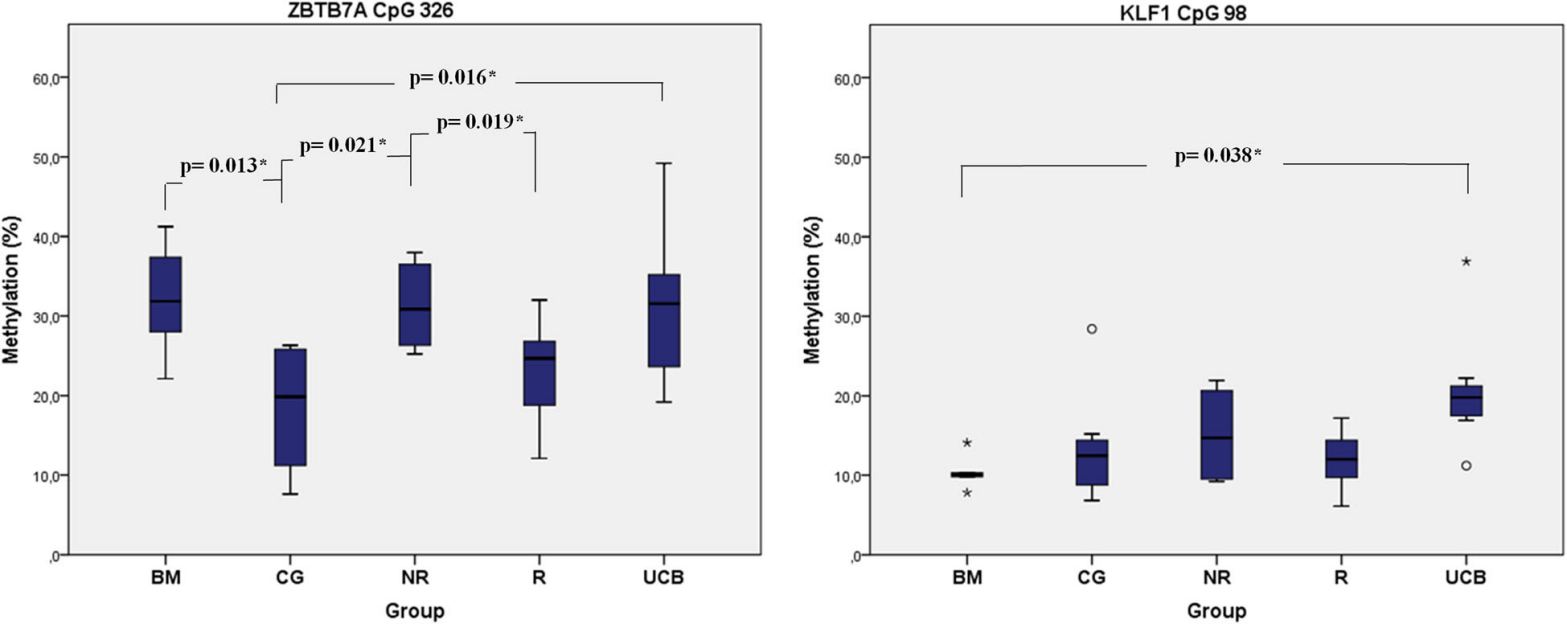
Mean methylation rate with statistical significant p* values of *ZBTB7A* and *KLF1* CpG sites in untreated single BFU-E colonies among different groups from Table 2 (in vitro samples). Data derived from single BFU-E colonies before the addition of HU are presented as boxplots. Boxes represent the 25th and the 75th percentiles of total values. They include the maximum and minimum values, the median value of the data set (lines inside the boxes), the most extreme values (*), the outliers (°), and the significant p* values. BM, CG, NR, R, and UCB indicate bone marrow, control group, HU non-responders, HU responders, and umbilical cord blood respectively

To the rest of the studied CpGs, the addition of HU (100 μΜ) caused a general mild decrease in CpG methylation status with minor exceptions. Differential baseline methylation patterns within the CpG islands were obtained, before the HU treatment, between the differently originated descendants (BFUEs: IV CG1–4, III R1–5, III NR1–4, IV UCB1–4, and IV BM1–4). This observation suggests that HU action exhibits epigenetic diversity towards individual primary cell types. Statistically significant differences in the baseline methylation levels were recorded from *ZBTB7A* CpG326 between both BM (p*, 0.013) and UCB (p*, 0.016) with CG and between NR with CG (p*,0.021) and NR with R (p*, 0.019). Similar results were obtained from *KLF1* CpG98 between BM and UCB (p*, 0.038) (Fig. 3).

**Fig. 4:**
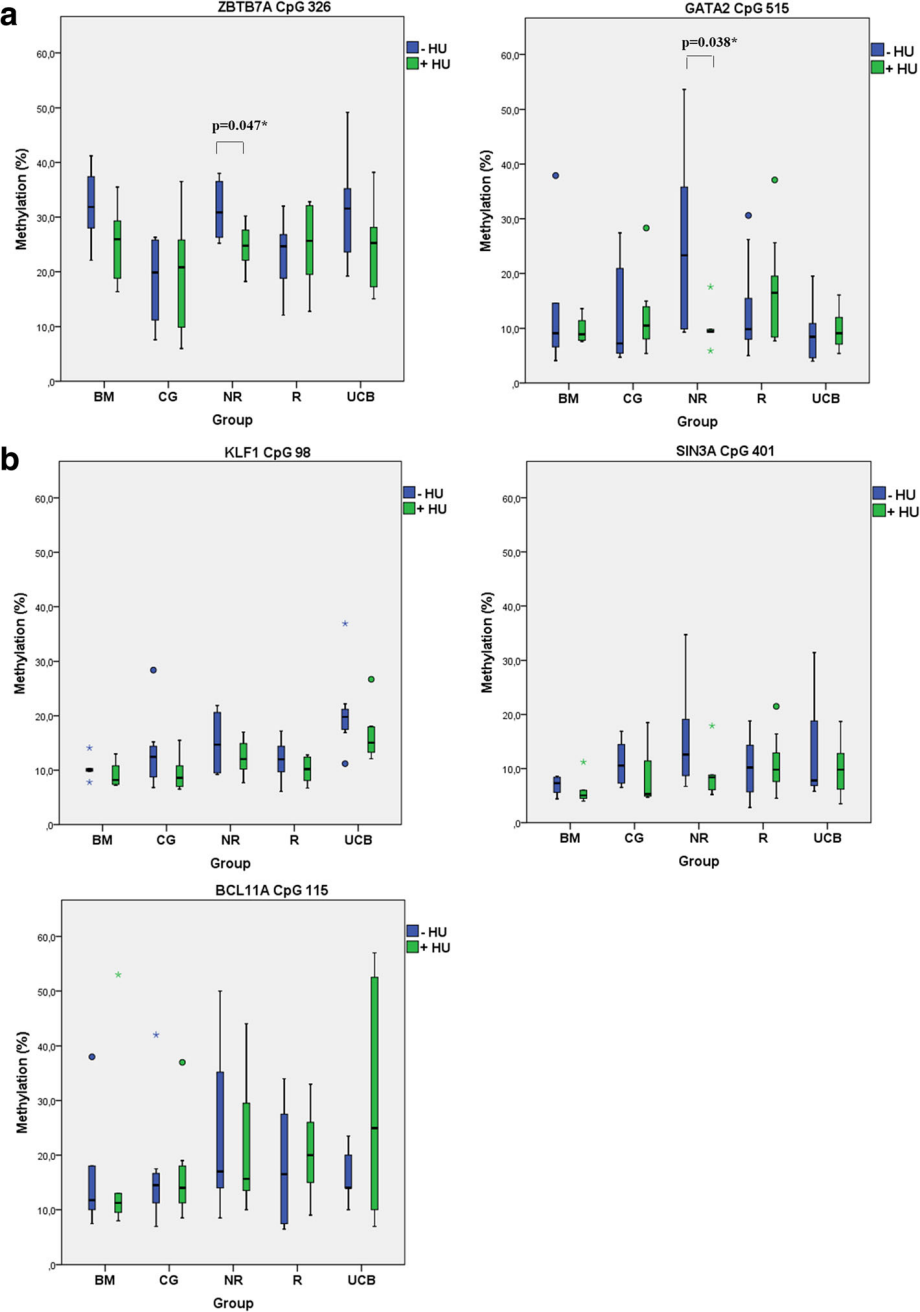
Mean methylation rate of CpG sites in single BFU-E colonies before and after HU treatment among all in vitro groups of samples (Table 2). **a** *ZBTB7A* CpG 326 and *GATA2* CpG 515 showed a significant hypomethylation profile in non-responders (NRs). **b** *SIN3A* CpG 401, *KLF1* CpG 98, and *BCL11A* CpG 115 showed hypomethylation trend in NRs, though not of statistical significance. Data are presented as boxplots. Boxes represent the 25th and the 75th percentiles of total values. They include the maximum and minimum values, the median value of the data set (lines inside the boxes), the most extreme values (*), the outliers (°), and the significant p* values recorded. BM, CG, NR, R, and UCB indicate BFU-Es derived from bone marrow, control group, HU non-responders, HU responders, and umbilical cord blood respectively

Statistically significant hypomethylation was obtained in the *ZBTB7A* (p*, 0.047) and *GATA2* (p*, 0.038) CpG islands only in the NR group of patients after HU treatment (Fig. 4a). BFU-Es derived from the non-responders showed the same trend of a mild hypomethylation profile in *KLF1*, *SIN3A*, and *BCL11A* CpGs, but of no statistical significance (Fig. 4b) (Additional file 1: Figure S2).

The hypomethylation status of the *ZBTB7A* CpG326 located at the gene’s proximal promoter region could lead to a possible elevated expression of LRF (leukemia/lymphoma-related factor) and the protein product of *ZBTB7A* gene and could have emerged the failure of γ-globin induction above the limit of the 2.1-fold (maximum observed γ-globin relative expression), on account of the erythropoietic cells’ exposure to the HU agent among non-responders. This observation led us to trace LRF/*ZBTB7A* expression levels among responders and non-responders.

### Detection and recording of LRF/*ZBTB7A* mRNA levels

**Fig. 5:**
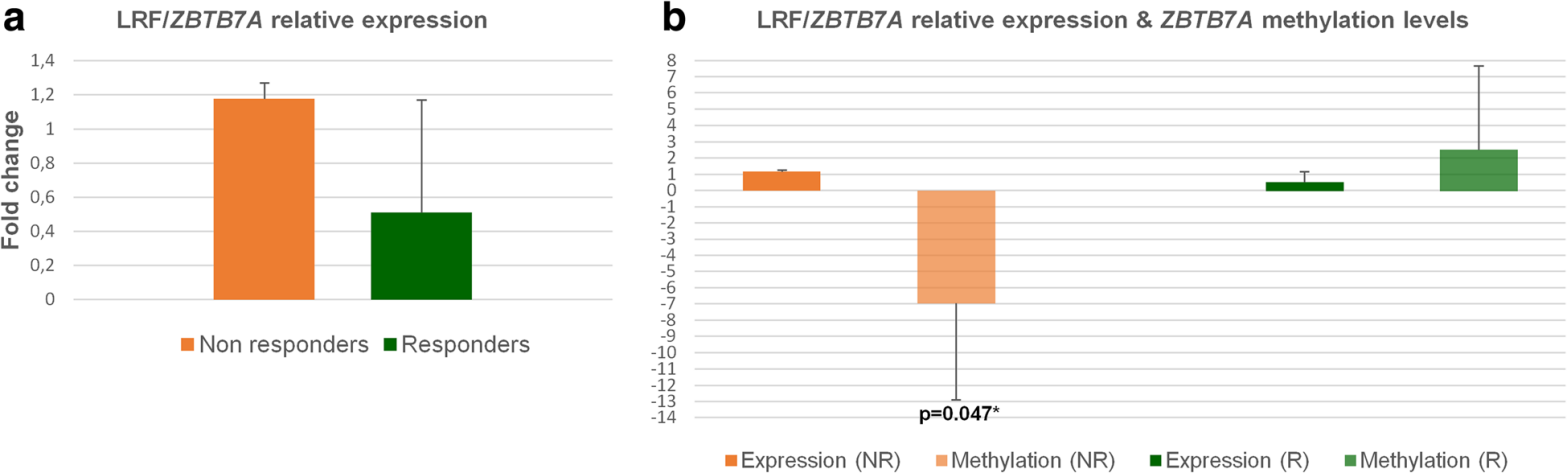
Relative expression of LRF/*ZBTB7A* and statistical correlations between gene’s methylation and expression. **a** Relative expression of LRF/*ZBTB7A* in BFU-E colonies of responders and non-responders (III R1–5, III NR1–4) after HU treatment. **b** Correlation between methylation and gene expression results in responders and non-responders (III R1–5, III NR1–4). Hypomethylation results in *ZBTB7A* CpG 326 were in line with the higher expression of *ZBTB7A* observed in non-responders. Opposite results were detected in the responders’ group

Specific primers to detect the LRF mRNA were designed (Table 3) and subjected to qPCR to quantitate its expression profile before and after the HU addition to cultivated cells. Total RNA for qPCR experiments was extracted from 30 to 50 characterized BFU-E colonies for each sample, to ensure that the LRF/*ZBTB7A* expression levels represent cells of the erythroid lineage and are consistent to similar methylation results obtained from single BFU-E colonies. LRF expression was calculated as previously described (“Materials and methods” section). Relative LRF expression was elevated only in NR group (1.1 to1.4-fold increase) in the presence of HU, which was in consistency with the hypomethylation results obtained in *ZBTB7A* CpG326 among the same group (NRs) (Fig. 5a, b). Similar studies delineate the expression of LRF/*ZBTB7A* at a more mature stage of red cell differentiation, after the proerythroblast stage [35]; hence, its expression at BFU-E colonies is at a minimum detectable level and only upon the simultaneous treatment of cells with the HU.

## Discussion

The fine regulation of the *HBB* cluster “modifier genes” caused by their natural epigenetic variations on DNA methylation profile and/or by HU medication widely used to treat β-hemoglobinopathies has been the target of investigation in the present study. CpG islands included in the study were the *BCL11A*, *KLF1*, *MYB*, *MAP3K5*, *SIN3A*, *ZBTB7A*, and *GATA2*, which are known to influence the natural developmental switch in hemoglobin expression, from fetal to adult. Nuclear factors and their genetic variations have long been investigated, but whether and to what extent they cooperate with epigenetic signals in fetal globin repression/induction is not yet fully understood.

The CpG sites selected for the present study bared factors involved in chromatin remodeling, such as the CCCTC-binding factor, known as *CTCF* and the leukemia/lymphoma-related factor (LRF/*ZBTB7A*). *CTCF* is a chromatin structural factor that plays a key role in gene expression through the activation or repression of gene promotes, the insulation of enhancers, and the regulation of distant chromatin interactions [36]. Binding of CTCF on DNA can be altered by DNA methylation. Especially, when CTCF-binding sites contain CpG dinucleotides, binding could be methylation specific, suggesting a role for *CTCF* in epigenetic regulatory mechanisms [37]. Our findings in respect to the methylation repertory of the *HBB* cluster “modifier genes” within the CpG regions encompassing CTCF-binding sites showed that it was normally distributed along the CpG island. These results indicated that the CTCF factor did not preserve any insulating function (at least for the genes studied) that could prevent the hypo- or hyper-methylation spreading across the specific CpG genome sequences, in the presence of HU.

The LRF/*ZBTB7A* is a member of the POK (POZ/BTB and Krüppel) protein family, which binds to CG-rich regions and recruits histone deacetylases to gene promoters leading to a closed chromatin conformation that prevents transcription [38]. The bioinformatics analysis showed that recognition binding sites for *CTCF* and *ZBTB7A* factors were settled at contiguous DNA sequences, while *ZBTB7A-*binding sites were apparent in all CpG areas selected for this study (Fig. 1). LRF/*ZBTB7A* factor has been characterized as an HbF repressor that acts independently from the known fetal globin repressor *BCL11A*, by recruitment of the NuRD complex [39, 40]. Another study has uncovered the combined action of *KLF1* with *ZBTB7A* to depress HbF [41].

Results from the in vitro methylation assays performed in this study clearly showed a hypomethylating trend in non-responders (III NR in Fig. 4) in the presence of HU, which reached a statistical significance for the *ZBTB7A* (−HU/+HU, p*, 0.047) and the *GATA2* (−HU/+HU, p*, 0.038) CpGs. Parallel gene expression experiments with RNA isolated from BFU-Es of the same cultures allowed us to directly correlate changes in methylation to gene expression. Hypomethylation of the *ZBTB7A* CpG326 was associated with higher levels of LRF/*ZBTB7A* expression in non-responders (III NR), up to 1.4-fold (Fig. 5). Hypomethylation of *GATA2* CpG515 potentially enhances the GATA2 protein expression too, but levels of GATA2 mRNA were not targeted in this study. The GATA2 high expression was considered assured as premature erythroid cells (and BFU-Es) are known to express higher levels of GATA2, which in later stages of maturation switches to GATA1 [28, 42].

The HU hypomethylation effect was also visible in *KLF1* CpG98, *SIN3A* CpG401, and *BCL11A* CpG115, but not at significant rates, whereas *MYB* CpG216, *MAP3K5* CpG172, and *BCL11A* CpG120 were methylated at nugatory values before and after HU. The detected minor changes in their methylation profiles were likely to be borderline, and thus, statistical analysis of these areas was not performed. Furthermore, the non-responder group of patients (III NR) had an established baseline higher methylation profile in CpG326 of *ZBTB7A*, in the absence of HU, compared to responders (III R) (p*, 0.019) and healthy donors (IV CG) (p*, 0.021) (Fig. 3), suggesting an underlying epigenetic mechanism of HU action by the methylation modulation of the *ΖΒΤΒ7Α*, leading to an amplitude modulation of its expression. The LRF encoded by the *ZBTB7A* gene preferentially binds methylated CpGs and acts as a γ-globin repressor [39]. This is in accordance with our results derived from the HU non-responders. Probably, their higher baseline methylation status along the *HBB* “modifier gene loci” favors the LRF binding, leading to a hardly irreversible suppression of the HbF production even under the HU influence.

Additionally, there were statistically significant differences in the CpG326 of *ZBTB7A* among IV CG and IV UCB (p*, 0.016) and IV CG and IV BM (p*, 0.013). Evidence from human systems indicates that pluripotent hematopoietic stem cells exhibit an epigenetic memory, related to their donor cell type of origin [43], and our results support these observations.

Results of the present study indicate that HU resets the methylation status and eventually the basal program of expression for several factors and definitely to loci unlinked to the *HBB* cluster. The HU non-responders demonstrated an extra barrier of epigenetic regulation for the γ-globin reactivation. Even though HU provoked a similar terminal hypomethylation status to both the HU non-responders and HU responders, (Fig. 4a), the γ-globin expression was not ultimately prevailed in the HU non-responders. The most likely explanation is that expression of the β- and γ-globin genes is still inherently unstable at the BFU-E stage and that micro-environmental conditions persisting during the erythroid differentiation determine whether β- or γ-globin expression will be favored.

Previous attempts to detect DNA methylation changes in CpG sequences lying at neighboring areas to the γ^G^-globin promoter have not achieved any informative insights [44]. Also, the HU epigenetic effect is manifested only in *HBB* modifier factors, derived from cells of the erythroid lineage, which was apparent from our study. A considerable heterogeneity on the epigenetic effect detected at the in vivo samples was attributed to the different origins and the subsistence of mixed cell populations within PBMCs. On the contrary, BFUEs (in vitro samples) consist of a cell population obtained from cell/tissue culture systems, uniformly differentiated towards the erythropoietic lineage, albeit PBMCs isolated from blood samples included a mixture of all mononuclear blood cells, which although they were exposed to the daily dose of HU, they seem not to have responded homogeneously. The methylation profile of all CpG islands gave significant results only when the research focused on well-characterized single erythroid colonies (BFU-Es), with the unique exception of the *SIN3A* CpG 401, which is not an erythroid tissue-specific factor. The SIN3A protein consists of multiple protein interaction domains and contacts a variety of binding partners to perform its biological function, whereas it is considered to co-operate with histone-modifying complexes exhibiting a global transcription regulatory property [32]. Hence, *SIN3A* was differentially methylated (p*, 0.024) between the in vivo samples (PBMCs) of HU non-responders and HU responders.

The role of *ZBTB7A* as an HbF repressor is fully supported by our data, due to its broad existence as a binding factor to all CpG islands selected and analyzed in this study, its significant hypomethylation, and the elevated levels (although modest) of LRF/*ZBTB7A* expression in non-responders treated with HU (Fig. 5). On the contrary, the baseline hypomethylated ZBTB7A-binding sites in the HU responder group of patients either do not show preference for the LRF binding or LRF is produced at adverse levels unable to force the γ-globin repression.

The present epigenetic analysis of the *HBB* cluster “modifier genes” provides some valuable information related to the heterogeneous response to HU, in an effort to predict patients’ response to treatment. We evaluated the HU potential as a hypomethylating agent although HU has not been previously associated with epigenetic responses, other than enhancing the expression of specific miRNAs [45–47]. Several miRNAs have been highlighted as epigenetic regulators of erythroid differentiation that influence HbF production (miR-26b, miR-151-3p, miR210) upon HU treatment. In contrast to fixed genome sequence, epigenetic patterns are plastic; thus, correcting aberrant, disease-causing epigenetic marks holds considerable therapeutic promise.

## Conclusions and future perspectives

A drawback to current HU treatment for the SCA and SCA/β-thal patients is its non-specific effects. Our pharmacoepigenomic study reveals the epigenetic pharmacological activity of HU distinguishes the different epigenetic regulatory nuclear mechanisms acting among HU responders and HU non-responders and contributes to the development of a clinical algorithm for improved prediction of the patients’ response to HU treatment.

The *ZBTB7A* appears as an emerging epigenetic regulator of the HbF induction bypassing the already known repressors of γ-globin expression. *ZBTB7A* possibly exerts its repressing function to the γ-globin gene through a chromatin remodeling pathway, which is supported by the broad existence of its multiple recognition binding sites close to all *HBB* cluster “modifier loci” tested. The *ZBTB7A*/LRF epigenetically induced binding to preconfigured *HBB* cluster “modifier genes” promoters should also be investigated, to uncover the vital changes to promoter accessibility and subsequent motivations for gene activation or repression.

Moreover, the methodology of the single-colony (BFU-E) DNA methylation analysis, developed by our lab, seems to be mostly appropriate and informative for the specific cell epigenetic responses towards the HbF re-induction pathway during treatment with HU. In addition to our results, it would be very interesting to investigate the epigenetic marks of reprogrammed erythroid progenitors further, under the influence of HU or other HbF inductive agents in immature proerythroblasts and other subpopulations of the hematopoietic cell lineage. Critical questions such as whether CpG islands of different tissues are uniformly targeted by the epigenetic machinery components or some are “safeguarded” by nuclear factors and remain unaffected need further insight.

Contemporary medication focused on the *HBB* locus-specific modifiers, used in conjunction with epigenetic biomarkers of response, as *ZBTB7A* CpG326 and *GATA2* CpG515, highlighted in this study, will enable truly precision interventions.

## Additional file

Additional file 1: **Table S1.** Hematological data of HU responders (R) and HU non-responders (NR) used as in vivo samples. Figure S1 In vivo HbF levels and γ-globin expression in vitro before and after HU treatment. (a) III NR2 and (b) III NR4 patients (Table 2) at baseline of HbF expression before HU treatment (c) III NR2, (d)III NR4 and (e) III R5 patients at the plateau phase, with maximum detected HbF expression levels. Arrows show the HbF curve. (f) γ-globin gene expression in BFU-E colonies calculated as relative expression of treated compared to untreated with HU colonies, using the formula 2^(-ΔCt)^ and normalized to the reference *GAPDH* gene. Figure S2 DNA methylation levels estimated by the pyrosequencing CpG assay. Typical pyrograms displaying DNA methylation analyses before and after cells’ treatment with HU of: (a) *ZBTB7A* CpG 326, (b*) GATA2* CpG 515, (c) *KLF1* CpG 98, (d) *SIN3A* CpG 401, (e) *BCL11A* CpG 115. Figure S3 DNA methylation levels less than 10% either with or without the addition of HU, estimated using the pyrosequencing CpG assay. Typical pyrograms displaying DNA methylation levels of (a) *BCL11A* CpG 120, and (b) *MYB* CpG 216. (ZIP 3179 kb)

## Abbreviations

BFU-E: Burst-forming unit-erythroid
BM: Bone marrow
CFU: Colony-forming unit assay
CFU-E: Colony-forming unit-erythroid
CG: Control group
HbF: Fetal hemoglobin
HU: Hydroxyurea
NR: Non-responders
PBMCs: Peripheral blood mononuclear cells
R: Responders
SCA: Sickle cell anemia
UCB: Umbilical cord blood
