# Supplementary figures and images for: Impact of ZBTB7A hypomethylation and expression patterns on treatment response to hydroxyurea
Source: Hum Genomics. 2018 Oct 1;12:45. doi: 10.1186/s40246-018-0177-z (PMC6167880; doi:10.1186/s40246-018-0177-z)

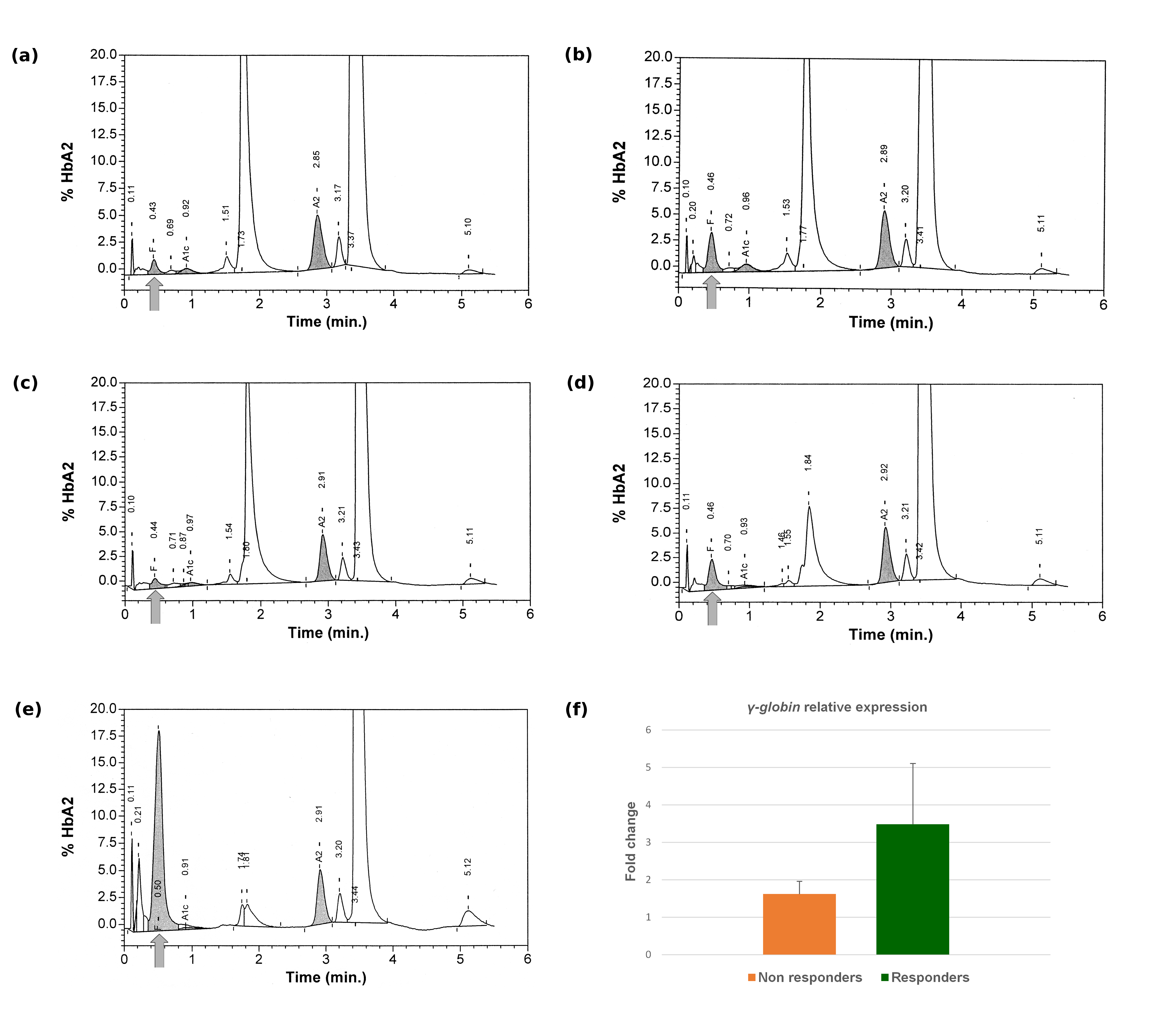

Supplement: Supplementary file 1 — Table S1. Hematological data of HU responders (R) and HU non-responders (NR) used as in vivo samples. Figure S1 In vivo HbF levels and γ-globin expression in vitro before and after HU treatment. (a) III NR2 and (b) III NR4 patients (Table 2) at baseline of HbF expression before HU treatment (c) III NR2, (d)III NR4 and (e) III R5 patients at the plateau phase, with maximum detected HbF expression levels. Arrows show the HbF curve. (f) γ-globin gene expression in BFU-E colonies calculated as relative expression of treated compared to untreated with HU colonies, using the formula 2(-ΔCt) and normalized to the reference GAPDH gene. Figure S2 DNA methylation levels estimated by the pyrosequencing CpG assay. Typical pyrograms displaying DNA methylation analyses before and after cells’ treatment with HU of: (a) ZBTB7A CpG 326, (b) GATA2 CpG 515, (c) KLF1 CpG 98, (d) SIN3A CpG 401, (e) BCL11A CpG 115. Figure S3 DNA methylation levels less than 10% either with or without the addition of HU, estimated using the pyrosequencing CpG assay. Typical pyrograms displaying DNA methylation levels of (a) BCL11A CpG 120, and (b) MYB CpG 216. (ZIP 3179 kb) [file 40246_2018_177_MOESM1_ESM.zip › FIGURE S1.tiff.tif]

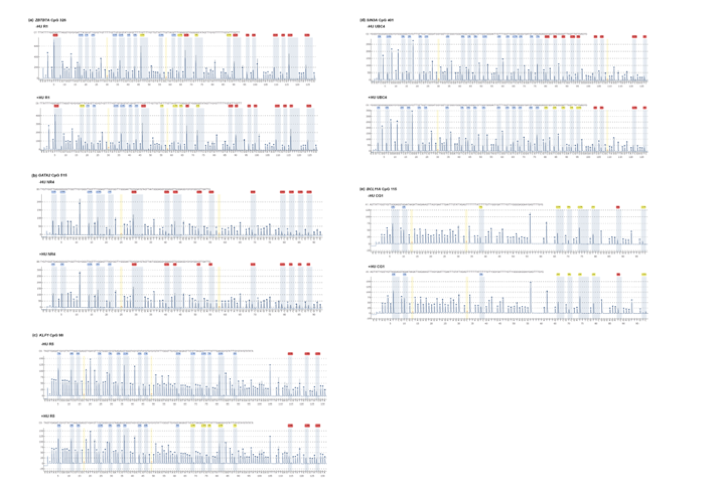

Supplement: Supplementary file 1 — Table S1. Hematological data of HU responders (R) and HU non-responders (NR) used as in vivo samples. Figure S1 In vivo HbF levels and γ-globin expression in vitro before and after HU treatment. (a) III NR2 and (b) III NR4 patients (Table 2) at baseline of HbF expression before HU treatment (c) III NR2, (d)III NR4 and (e) III R5 patients at the plateau phase, with maximum detected HbF expression levels. Arrows show the HbF curve. (f) γ-globin gene expression in BFU-E colonies calculated as relative expression of treated compared to untreated with HU colonies, using the formula 2(-ΔCt) and normalized to the reference GAPDH gene. Figure S2 DNA methylation levels estimated by the pyrosequencing CpG assay. Typical pyrograms displaying DNA methylation analyses before and after cells’ treatment with HU of: (a) ZBTB7A CpG 326, (b) GATA2 CpG 515, (c) KLF1 CpG 98, (d) SIN3A CpG 401, (e) BCL11A CpG 115. Figure S3 DNA methylation levels less than 10% either with or without the addition of HU, estimated using the pyrosequencing CpG assay. Typical pyrograms displaying DNA methylation levels of (a) BCL11A CpG 120, and (b) MYB CpG 216. (ZIP 3179 kb) [file 40246_2018_177_MOESM1_ESM.zip › Figure S2.tiff]

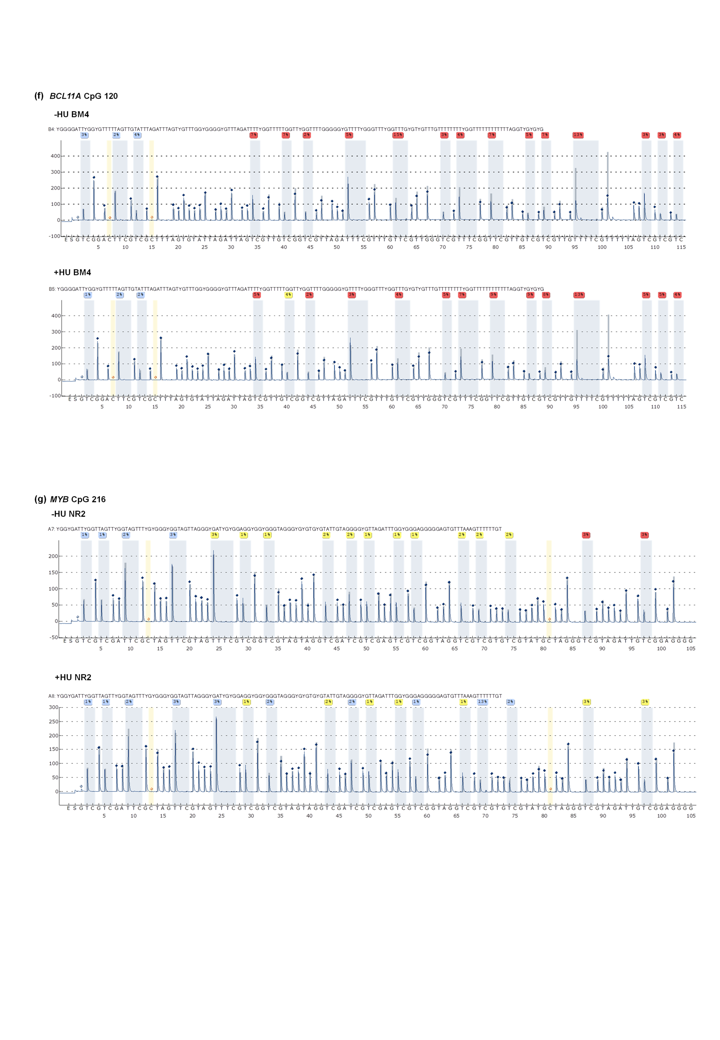

Supplement: Supplementary file 1 — Table S1. Hematological data of HU responders (R) and HU non-responders (NR) used as in vivo samples. Figure S1 In vivo HbF levels and γ-globin expression in vitro before and after HU treatment. (a) III NR2 and (b) III NR4 patients (Table 2) at baseline of HbF expression before HU treatment (c) III NR2, (d)III NR4 and (e) III R5 patients at the plateau phase, with maximum detected HbF expression levels. Arrows show the HbF curve. (f) γ-globin gene expression in BFU-E colonies calculated as relative expression of treated compared to untreated with HU colonies, using the formula 2(-ΔCt) and normalized to the reference GAPDH gene. Figure S2 DNA methylation levels estimated by the pyrosequencing CpG assay. Typical pyrograms displaying DNA methylation analyses before and after cells’ treatment with HU of: (a) ZBTB7A CpG 326, (b) GATA2 CpG 515, (c) KLF1 CpG 98, (d) SIN3A CpG 401, (e) BCL11A CpG 115. Figure S3 DNA methylation levels less than 10% either with or without the addition of HU, estimated using the pyrosequencing CpG assay. Typical pyrograms displaying DNA methylation levels of (a) BCL11A CpG 120, and (b) MYB CpG 216. (ZIP 3179 kb) [file 40246_2018_177_MOESM1_ESM.zip › Figure S3.tiff]
